# Parental decision and intent towards COVID-19 vaccination in children with asthma: an econometric analysis

**DOI:** 10.1186/s12889-022-13933-z

**Published:** 2022-08-13

**Authors:** Olivier Drouin, Pierre Fontaine, Yann Arnaud, Claude Montmarquette, Alexandre Prud’homme, Roxane Borgès Da Silva

**Affiliations:** 1 Division of General Pediatrics, Department of Pediatrics, CHU Sainte-Justine, 3175 chemin de la Côte-Sainte-Catherine, Montréal, QC H3T 1C5 Canada; 2 Department of Social and Preventive Medicine, School of Public Health, University of Montreal, 7101 avenue du Parc, Third floor (Office 3029), Montréal, QC H3N 1X9 Canada; 3 Faculty of Medicine, University of Montreal, 2900 boulevard Edouard-Montpetit (Pavillon Roger-Gaudry), Montréal, QC H3T 1J4 Canada; 4 CIRANO (Centre Interuniversitaire de Recherche en Analyse Des Organisations), 1130 rue Sherbrooke Ouest #1400, Montréal, QC H3A 2M8 Canada; 5 Department of Management, Evaluation and Health Policy, School of Public Health, University of Montreal, 7101 avenue du Parc, Third Floor (Office 3076), Montréal, QC H3N 1X9 Canada

**Keywords:** COVID-19, Vaccine, Asthmatic children, Parental decisions, Econometric modeling

## Abstract

**Objective:**

Vaccination will be instrumental in controlling the COVID-19 pandemic, and vaccination of children will be necessary to achieve herd immunity. Given that children with chronic health conditions may be at increased risk of COVID-19, it is crucial to understand factors influencing parental decisions about whether to have their child vaccinated. The study objectives were to measure parental intent to have their child with asthma vaccinated against COVID-19 and identify the determinants of their vaccination decision.

**Study design:**

This study is based on a cross-sectional exploratory observational online survey assessing parents' risk perception in the context of COVID-19.

**Methods:**

In this study conducted in August 2020, the primary outcome was parent’s answer to the question on their intention to get their child vaccinated if a vaccine against COVID-19 was available. Participants were also asked about their intention to get vaccinated themselves. Independent variables studied included sociodemographic, clinical data (e.g. presence of other chronic diseases), psychological, cognitive and risk perception related to COVID-19. Simultaneous equations models (3SLS) and seemingly unrelated regressions model (SUR) were carried out to identify factors associated with intention to have the child vaccinated and participants’ intention to get vaccinated themselves against COVID-19.

**Results:**

A total of 305 participants completed the survey. Overall, 19.1% of participants reported being unlikely or very unlikely to vaccinate their child against COVID-19 if a vaccine was available. Similarly, 21.0% were unlikely or very unlikely to get vaccinated themselves. The following factors were significantly associated with parents’ decision to have their child vaccinated: parental level of education (*p* = 0.003), employment status (*p* < 0.001), sex of the child (*p* = 0.019), presence of other chronic diseases (*p* = 0.028), whether or not the child had been vaccinated against influenza in the past (*p* < 0.001), parental anxiety (*p* = 0.046), and consultation with a health professional since the beginning of the pandemic (*p* = 0.009). There was a strong relationship between likelihood of not intending to have one’s child vaccinated and personal intent not to get vaccinated.

**Conclusion:**

These findings are essential in planning for the communication and dissemination of COVID-19 vaccination information to parents, especially for children with asthma or other chronic medical conditions.

**Supplementary Information:**

The online version contains supplementary material available at 10.1186/s12889-022-13933-z.

## Background

As the COVID-19 pandemic spreads around the world, much hope and effort have been invested in finding an effective vaccine [1]. However, developing an effective COVID-19 vaccine is only a first step in achieving immunity and controlling the pandemic [2]. Since several effective vaccines are now available for adults, but also adolescents and school-age children, it has become clear that acceptance of vaccination by the general public remains a sine qua non element of any effective vaccine rollout strategy [3–5]. Worryingly, numerous reports suggest that people’s willingness to get vaccinated against COVID-19 is far from universal.

Historically, vaccination has been one of the safest and most cost-effective public health interventions available [6–9]. Children’s routine vaccinations, including against pneumococcus, polio, and measles, are public health success stories. A large majority of people accept children’s routine immunization. Yet, there is significant variation in vaccine acceptance, owing to a variety of vaccine-related and individual factors, which may have relevance to a COVID-19 vaccine for children [10].

Among existing vaccines, more recently developed ones, such as the human papillomavirus vaccine, have lower acceptance rates and are more prone to generate vaccine hesitancy [11]. Similarly, vaccines requiring yearly administration or those of uncertain effectiveness (such as the annual influenza vaccine) have lower rates of uptake [12]. In contrast, the perception of a vaccine as being part of routine vaccination, instead of part of a specific vaccination campaign, leads to greater acceptance [13].

### COVID-19 vaccination acceptance

As of January 2022, 60.5% of the world population has received at least one dose of a COVID-19 vaccine [14]. Vaccination rates continue to lag in low-income countries, where only 10 percent of the population has received at least one dose of a vaccine, whereas in high- and upper-middle-income countries, 77 percent of the population has received at least one dose [14]. The cumulative percent of people who have received at least one dose of a COVID-19 vaccine in Canada is 83.4% with variation between provinces (lowest in Nunavut at 78.5% and highest in Newfoundland and Labrador at 93.8%) [15]. Factors associated with vaccination against COVID-19 are still the subject of a variety of investigations around the world. In Canada, vaccination coverage is highest in adults aged 70 and older (≥ 95% two doses vaccinated) and a greater percentage of females than males have receives two doses (78.8% in female versus 75.9% in male)[15]. In general, greater acceptance is found among women, older adults, those with higher education, and those with greater trust in government [16, 17]. Positive opinions towards public sector officials and positive attitudes about safety and effectiveness of the vaccine are also associated with vaccine uptake [18, 19].

### Determinants of child vaccination against COVID-19

Before COVID-19, factors associated with willingness to have one’s child vaccinated had seldom been studied in the context of a pandemic. Early studies by Goldman et al. based on cross-sectional surveys showed that less than 50% of parents were be willing to have their child vaccinated against COVID-19 [20]. In jurisdiction where COVID-19 vaccines have been approved for children, there was lower uptake for children than older age groups. Even this finding should be nuanced as there are important variations in COVID-19 vaccination coverage in children across different segments of the population. A recent study from a cohort study in Montreal, Canada, shows that children from households with annual incomes < $100,000 had 18.4 percentage point lower chance of being vaccinated compared to household incomes ≥ $150,000 (95% CI: 10.1 to 26.7). The study also reveals, vaccine-eligible adolescents from the most deprived neighbourhood were half as likely to be vaccinated compared to those from the least deprived neighbourhood (aPR = 0.48; 95% CI: 0.18 to 0.77) [21].

Early in the pandemic, a study conducted with a convenience sample of parents in England showed that most parents said they would likely accept a COVID-19 vaccine, both for themselves and for their children. In that study, visible minorities were less likely to report wanting to be vaccinated against COVID-19, as were participants of lower socioeconomic status. However, the study did not systematically examine mechanistic explanations for the differences between sociodemographic groups. In open-text responses and interviews, the primary motivation given for getting vaccinated was self-protection, while concerns regarding a rapidly developed vaccine’s safety were a predominant worry [22].

In a study of children presenting to pediatric emergency departments from March to May 2020, 65% of caregivers reported that they intended to have their child vaccinated against COVID-19 [20]. Determinants of a higher likelihood of reporting intent to vaccinate were older children, children without chronic disease, recent history of influenza vaccination, and caregivers’ concerns about COVID-19 [20].

Finally, regarding parents’ acceptance of a COVID-19 vaccine for themselves, an Australian study conducted during the first wave of the pandemic showed that 16.7% of parents were unsure, and 7.6% were unwilling to accept a COVID-19 vaccine. Of those, the vast majority were concerned about vaccine efficacy and safety, while one in four believed that the vaccine was unnecessary [23].

At the time when the current study was conducted (August 2020), COVID-19 vaccines for children were still being developed. As of January 2022, vaccination against COVID-19 was available for children 5–11 years and adolescents in Canada and the U.S., but was not yet available in younger children [24]. In lower-income countries, vaccination for children and adolescents may still only be considered. Therefore, even today, it is important to better understand the factors that may have an impact on the vaccine intention of parents in relation to their children.

### Vaccination in children with asthma

Asthma is a common chronic respiratory disease, affecting one in ten children, making it one of the most common chronic diseases of childhood [25, 26]. Presence of a chronic disease is a recognized risk factor for more severe disease among children hospitalized for COVID-19 [27]. Generally, children with asthma tend to have a more severe respiratory virus infection presentation, especially among those with poorly controlled disease. A recent study revealed that children and young people aged 5–17 years with poorly controlled asthma are at increased risk of hospitalization due to COVID-19 [24]. As such, by both their increased risk of severe disease and by representing the largest group of children with chronic condition, children with asthma are an important yet understudied group with regards to COVID-19 vaccine hesitancy.

Vaccine hesitancy studies in children with asthma have been conducted regarding influenza vaccination, showing that children were more likely to be vaccinated if they were younger, if parents believed the vaccine had good efficacy, and if parents had few worries about potential side effects. Interestingly, asthma control level did not appear to be a significant factor in parents’ decision to vaccinate [28]. Importantly, children were much more likely to get vaccinated if the vaccine had been recommended by a physician [29]. Studies performed during the H1N1 pandemic also showed that parent-reported intent to vaccinate among children with asthma was low, with no effect of asthma control. Still, prior vaccination for influenza and beliefs and attitudes regarding the influenza A/H1N1 vaccine were significant determinants of their decision [30]. In this context, physician recommendation was a decisive factor influencing intent to have a child vaccinated. To date, we are not aware of any study examining determinants of parents’ decision to have their child with asthma vaccinated against COVID-19.

### Behavioral economics

To ensure that large-scale COVID-19 vaccination efforts are successful and to guarantee vaccine uptake, it is necessary to go beyond sociodemographic characteristics and understand the determinants of people’s decisions to get vaccinated. Differences in acceptance between age groups or across socioeconomic statuses are likely due to other factors, such as risk perception, numeracy, or risk tolerance. It is essential to understand those other factors, given that, as opposed to age and sex, they are malleable and amenable to intervention. Behavioral economics examines determinants of behaviors beyond expected utility and can help us understand people’s decisions made under uncertainty. It can also help us comprehend difficult and puzzling behaviors, such as vaccine hesitancy and vaccine refusal. Beyond its descriptive capacity, the field can also shed light on important potential interventions to encourage socially desirable behaviors like vaccination [31, 32]. The importance of understanding behavioral aspects of COVID-19 vaccination has been recognized by the World Health Organization, which recently published a technical report on “Behavioral Considerations for Acceptance and Uptake of COVID-19 Vaccines” [33].

### Objectives

The objectives of this study conducted in the summer of 2020 were to measure parental intent to have their child with asthma vaccinated against COVID-19 and identify the determinants of their vaccination decision.

## Methods

### Study population and data collection

The participants in this study were parents of children with asthma, followed in a specialized asthma clinic of a pediatric tertiary care center of Montreal, Canada. Only parents that had previously indicated to the asthma clinic their interest in participating in studies were invited to participate to the study (*n* = 580).

This study used a de-identified online cross-sectional survey conducted between July 30 and August 17, 2020. All potential participants were first invited by email to complete the survey via a secure and personalized hyperlink leading to online questionnaire (Lime Survey). Up to two reminders were sent by email in the following 10 days.

The ethics committee of the CHU Sainte-Justine has approved the study and the data collection procedures (# 2021–3032). The informed consent (electronic consent) of participants was obtained before completing the online questionnaire. All methods were performed in accordance with the relevant guidelines and regulations.

### Dependent variables

#### Intention to get vaccinated against COVID-19

Our primary dependent variable was parents’ stated intention to have their child vaccinated, in response to the following question: “If a vaccine for COVID-19 was available today, what is the likelihood that you would have your child vaccinated?” Participants answered on a 4-point Likert scale ranging from Very Unlikely to Unlikely, Likely, and Very Likely, with a response option for “I don’t know or refuse to answer.” As a secondary outcome, we also asked parents if they themselves intended to get vaccinated, using the same answer categories.

### Independent variables

#### Sociodemographic

Sociodemographic variables included the parent’s sex, age, level of education, work status, and region of residence. The sex and age of the child were also covariates.

#### Clinical

Participants were asked to report on their child’s asthma control using the validated Asthma Control Test that has been used in other COVID studies [34–36]. We enquired about consulting with a physician or health professional at the onset of the pandemic and whether the child had been vaccinated against influenza in the previous year. We also asked parents whether their child had any other chronic medical conditions [37].

#### Psychological

We evaluated participants’ personal worries during the COVID-19 pandemic with the Lavoie and Bacon survey questionnaire on COVID-19 Awareness and Responses [38]. Participants’ anxiety was measured using the General Anxiety Disorder (GAD-7) scale, a short validated scale used in other COVID-related publications [35].

#### Risk perception

To understand how the risk of COVID-19 is perceived, we asked parents what they thought was the likelihood that their child would be infected with COVID-19 in the coming months. Parents were also asked about their perceived level of control in preventing COVID-19 infection in their child [39]. Finally, parents were asked if they knew someone who had been infected with COVID-19.

#### Cognitive

Participants were administered Frederick’s Cognitive Reflection Test [40]. The test measures an individual’s ability to think “slow” rather than “fast,” using the terminology of Kahneman [41]. Individuals who read and answer quickly (fast thinking) are less likely to answer correctly. Participants were also asked to complete Jappelli’s numeracy test [42].

### Model specifications

The survey data have been analyzed with appropriate econometric models. Causality issues are discussed, with a path analysis of parents’ decisions to vaccinate their child and themselves. Bivariate analysis prior to regression models was executed to analyze the correlations between the independent variables themselves, between the dependent variables themselves, and between the independent and dependent variables. To determine the strength of the associations, we used Cramer's v and Pearson coefficient.

The relationship between the two dependent variables (intent to have the child vaccinated, and parents’ intent to get vaccinated themselves), along with the proper independent variables for both equations suggests that different econometric models need to be explored to account for the causality issue.

As mentioned earlier, both dependent variables are measured using a 4-point Likert scale, with a response option for “I don’t know or refuse to answer.” We kept the individuals in our sample that chose this last answer and opted for a 5-point Likert scale ordered in the following way: I do not know or refuse to answer, unlikely, very unlikely, likely, very likely.^1^

**Fig. 1:**
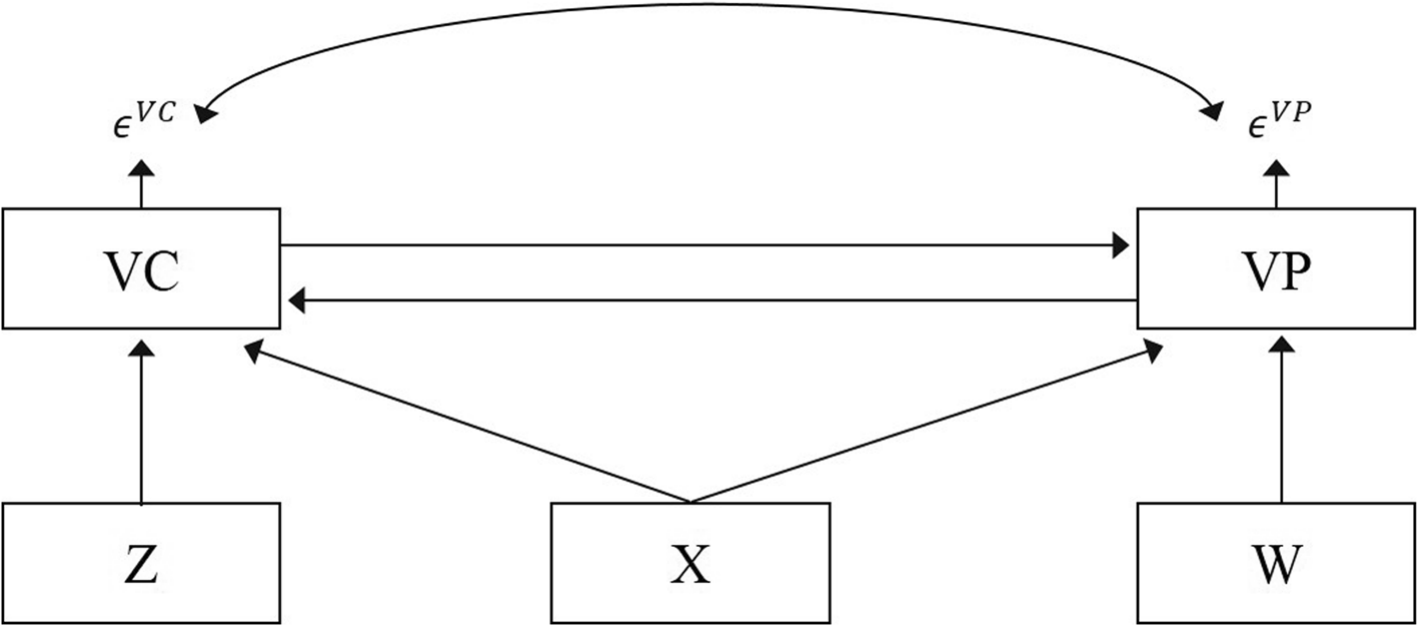
Path analysis of parents’ intentions to have their child and themselves vaccinated

Figure 1 illustrates the different causality issues involved in parents’ intention to have their children and themselves vaccinated against COVID-19, emphasizing the directions of causation.

The general model is a simultaneous equations model between VC (vaccination of a child) and VP (vaccination of a parent), one influencing the other and vice-versa and with correlated error terms ${\epsilon }^{VC}$ and ${\epsilon }^{VP}.$ X is a set of exogenous variables shared by both decisions. Z and W are sets of exogenous variables affecting the VC decision only and the VP decision only. Three-stage least squares (3SLS) will estimate this general model by linearizing the five categories of the dependent variables in 0,1,2,3,4 instead of keeping their qualitative ordering.

Other causality hypotheses are obtained by removing some arrows between the dependent variables. For example, considering the causality running from the decision to vaccinate a child as an explanation for the parents’ decision to accept a vaccine for themselves will remove the arrow in the other direction leading to a recursive model. Here, a two-step procedure will first run an ordered probit on a parent’s decision to vaccinate a child and then use the predicted values (in linear terms) to explain the decision to accept a vaccine for herself or himself. If causality runs in the other direction, the arrow from VC to VP is removed. The model is estimated with a two-step procedure as described above. Finally, supposing the two vaccination decisions are independent, then we estimate the model with a seemingly unrelated regressions model (SUR) to maintain the hypothesis that the error terms are correlated. Here, we need to linearize the five categories of the dependent variables in 0,1,2,3,4, as for the 3SLS model.

While the 3SLS model nests the SUR specification, the other models are not nested. The goal of estimating all four models is to come up with robust results relative to the exogenous variables. Our preferred model is presented in detail in the text, and one model is shown in the Appendix: Table A1). The results of the other models are briefly discussed (all estimates are available on request).

The usual statistics such as the Log pseudo likelihood and the pseudo R2, the Wald Test and the Chi-squared were calculated to assess the goodness of the model. RMSE (root-mean-square error) was used to assess the strength of our SUR model.

## Results

### Descriptive and summary statistics

In total, 305 participants completed the survey among the 580 contacted, for a response rate of 52.6%.

Table 1 shows that 63% of parents are likely or very likely to have their child vaccinated, and 64% are likely to get themselves vaccinated.

**Table 1:** Distribution (%) of parents’ answers about the likelihood of accepting a vaccine for their child and themselves (*n* = 305)

	I don’t know	Very unlikely	Unlikely	Likely	Very likely
Parents' intention to have their asthmatic child vaccinated against COVID-19	17.0	12.5	6.6	19.7	44.3
Parents' intention to have themselves vaccinated against COVID-19	15.1	14.1	6.9	18.4	45.6

Table 2 indicates that most of our participants were mothers (94.4%). With 68.5% of participants reporting a university level of education, our sample, while biased, concerns a segment of the population more likely to be better informed about vaccination.

**Table 2:** Summary statistics (%) of the independent variables (*n* = 305)

			%
**Sociodemographic characteristics**	Sex of parent	Male	5.6
		Female	94.4
	Age of parent	Under 35	21.3
		35 to 44	55.7
		45 and over	23.0
	Level of education	Secondary or less	18.7
		College (CEGEP)	12.8
		University	68.5
	Employment status	Inactive	20.7
		Active	79.3
	Region of residence	Montreal	52.5
		Other region	47.5
	Sex of child	Male	59.0
		Female	41.0
	Age of child	5 and under	26.6
		6 to 10	43.6
		11 and over	29.8
**Clinical characteristics**	Perceived control of child’s asthma	Less controlled	15.7
		More controlled	84.3
	Child has another chronic disease	No	94.8
		Yes	5.2
	Child was vaccinated against influenza last year	No	69.8
		Yes	30.2
	Consultation with a health professional	No	31.1
		Yes	68.9
**Risk perception**	Level of general anxiety	Lower	63.3
		Average	25.2
		Higher	11.5
	Level of concern regarding COVID-19	Lower	9.5
		Average	35.4
		Higher	55.1
	Perceived control of child’s risk of infection with COVID-19	Less controlled	37.0
		More controlled	63.0
	Know someone that has been affected by COVID-19	No	47.9
		Yes	52.1
	Perceived risks of infection with COVID-19	50%	39.7
		Less than 50%	38.0
		More than 50%	22.3
**Cognitive characteristics**	Numeracy level	Lower	60.3
		Higher	39.7
	Cognitive skill	Lower	74.8
		Higher	25.2
	Risk tolerance	Aversion	77.0
		Propensity	23.0

### Econometric results

The general model estimated by 3SLS stressed the strong relationship between the two decisions concerning vaccination (results not shown).^2^ However, only two exogenous variables were statistically significant: living in the Montreal region and parents with a high numeracy level. Keeping the causality in both directions among the decision variables rendered the other determinants meaningless.

The SUR model eliminated the direct relationship between the two decisions except for the error terms. It yielded information on the role of the exogenous variables. For the decision concerning whether or not to have a child vaccinated, the coefficient estimates of the following exogenous variables had a positive effect and were statistically significant at the 5% level or better: the parent reports a university level of education, is currently active in the labor market, and the child was vaccinated for the flu last year. With a 6.8% level of significance, parents that had consulted with a health professional also had a higher probability of accepting the COVID-19 vaccine for their child.

For the decision about whether or not to get vaccinated themselves, parents with a university level of education, currently active in the labor market, and with a high numeracy level had a higher probability of accepting the COVID-19 vaccine. With a 6.3% level of significance, parents expressing a high level of concern regarding COVID-19 also had a higher probability of accepting a COVID-19 vaccine. Note that SUR estimated the correlation between the error terms at 0.852.

Next, we consider a single causality direction between the independent variables with recursive two-step procedure models (the two-step procedure accounts for correlated error terms). One model assumes the direction of causality running from the decision to have the child vaccinated as the causal factor to explain their own decision to be vaccinated. A second model reverses the direction of causality. Both models are estimated with ordered probit using the 5-point Likert scale.

In light of the previous results and those of the next model, Table 3 presents the results for our preferred models, assuming that the decision regarding the child explains the parents’ vaccination decision.

**Table 3:** Recursive ordered probit models (child → parents)

			Child’s model		Parent’s model	
			Coef	p	Coef	p
**Parents’ intention that their child be vaccinated against COVID** (linear prediction)			Not included		0.965	< 0.001
**Sociodemographic characteristics**	**Sex of parent** (ref.: Male)	Female	0.431	0.110	-0.164	0.583
	**Age of parent** (ref.: Under 35)	35 to 44	0.040	0.806	0.024	0.880
		45 and over	-0.178	0.446	-0.103	0.602
	**Level of education** (ref.: Secondary or less)	College (CEGEP)	0.175	0.460	0.074	0.759
		University	0.563	0.003	0.069	0.728
	**Employment status** (ref.: Inactive)	Active	0.603	0.001	-0.017	0.931
	**Region of residence** (ref.: Other regions)	Montreal	0.217	0.122	0.134	0.333
	**Sex of child** (ref.: Male)	Female	0.341	0.019	Not included	
	**Age of child** (ref.: 5 and under)	6 to 10	0.030	0.869	Not included	
		11 and over	0.368	0.100		
**Clinical characteristics**	**Perceived control of child’s asthma** (ref.: Less controlled)	More controlled	0.072	0.726	Not included	
	**Child has another chronic disease** (ref.: No)	Yes	0.565	0.028	Not included	
	**Child was vaccinated against influenza last year** (ref.: No)	Yes	0.789	< 0.001	Not included	
	**Consultation with a health professional** (ref.: No)	Yes	0.405	0.009	Not included	
**Psychological**	**Level of general anxiety** (ref.: Lower)	Average	-0.038	0.833	-0.089	0.610
		Higher	0.493	0.046	-0.092	0.705
	**Level of concern regarding COVID-19** (ref.: Lower)	Average	0.324	0.147	-0.140	0.514
		Higher	0.099	0.654	0.033	0.878
**Risk perception**	**Know someone that has been affected by COVID** (ref.: No)	Yes	Not included		0.021	0.883
	**Perceived control of child’s risk of infection with COVID-19** (ref.: Less controlled)	More controlled	0.033	0.813	Not included	
	**Perceived risks of infection with COVID-19** (ref.: 50%)	Less than 50%	-0.217	0.168	Not included	
		More than 50%	-0.101	0.601		
**Cognitive characteristics**	**Numeracy level** (ref.: Lower)	Higher	0.208	0.204	0.127	0.439
	**Cognitive skill** (ref.: Lower)	Higher	0.077	0.667	-0.020	0.913
	**Risk tolerance** (ref.: Aversion)	Propensity	0.209	0.171	-0.043	0.780
	Log pseudolikelihood		-384		-381	
	Pseudo R2		0.114		0.117	

In this model, the decision about whether to vaccinate a child is assumed to depend on exogenous factors only (the first step in the procedure). Seven variables present coefficient estimates reaching statistical significance at the level of 5% or better and with their expected sign: parental education, employment status, sex of the child, presence of other chronic diseases, child had been vaccinated against influenza in the past, parental anxiety, and consultation with a health professional since the beginning of the pandemic. We note that four of those variables were identified with the SUR model as statistically significant for robustness.

The probability that a parent will agree to be vaccinated (parents’ model, second step) is exclusively explained by the predicted values (linear predictions) of their decision to have their child vaccinated.

Table 4 illustrates the factors influencing the predicted parents’ intention that their child with asthma be vaccinated against COVID-19 based on that model.

**Table 4:** Predicted distributions (%) of parents’ intention to have their asthmatic child vaccinated against COVID-19

			I don’t know	Very unlikely	Unlikely	Likely	Very likely
			Pr(IF_i_ = 0)	Pr(IF_i_ = 1)	Pr(IF_i_ = 2)	Pr(IF_i_ = 3)	Pr(IF_i_ = 4)
**Sociodemographic characteristics**	Sex of parent	Male	34.5	15.9	7.4	18.1	24.0
		Female	16.0	12.0	6.7	20.0	45.4
	Age of parent	Under 35	17.2	12.3	6.8	20.0	43.6
		35 to 44	14.4	11.2	6.4	19.7	48.4
		45 and over	23.6	14.2	7.3	19.9	35.0
	Level of education	Secondary or less	36.4	16.6	7.6	18.1	21.3
		College (CEGEP)	26.4	15.4	7.7	20.2	30.3
		University	10.7	10.0	6.0	19.7	53.6
	Employment status	Inactive	38.6	16.4	7.4	17.3	20.4
		Active	12.4	10.7	6.3	19.7	50.9
	Region of residence	Other regions	21.5	13.8	7.2	20.0	37.4
		Montreal	13.2	10.9	6.3	19.6	50.0
	Sex of child	Male	19.6	13.3	7.1	20.3	39.7
		Female	12.8	10.7	6.2	19.6	50.7
	Age of child	5 and under	19.5	13.2	7.0	20.1	40.2
		6 to 10	18.9	13.0	7.0	20.1	41.1
		11 and over	12.3	10.4	6.1	19.2	52.0
**Clinical characteristics**	Perceived control of child’s asthma	Less controlled	17.9	12.7	6.9	20.1	42.3
		More controlled	16.5	12.2	6.8	20.1	44.5
	Child has another chronic disease	No	17.2	12.5	6.9	20.3	43.1
		Yes	7.8	8.0	5.0	17.6	61.6
	Child was vaccinated against influenza last year	No	20.6	14.3	7.7	21.4	36.0
		Yes	6.7	7.5	4.9	17.9	63.0
	Consultation with a health professional	No	22.7	14.3	7.4	20.4	35.2
		Yes	13.9	11.3	6.5	20.1	48.2
**Risk perception**	Level of general anxiety	Lower	18.5	12.8	6.9	20.1	41.7
		Average	19.8	13.2	7.1	20.1	39.8
		Higher	5.9	6.5	4.3	15.8	67.5
	Level of concern regarding COVID-19	Lower	26.7	15.1	7.6	19.8	30.8
		Average	13.2	10.9	6.3	19.8	49.8
		Higher	17.2	12.5	6.9	20.4	43.0
	Perceived control of child’s risk of infection with COVID-19	Less controlled	17.2	12.4	6.8	20.1	43.5
		More controlled	16.6	12.2	6.8	20.0	44.5
	Perceived risks of infection with COVID-19	50%	19.0	13.1	7.0	20.2	40.7
		Less than 50%	14.7	11.5	6.5	19.9	47.4
		More than 50%	16.6	12.3	6.8	20.1	44.2
**Cognitive characteristics**	Numeracy level	Lower	20.8	13.9	7.4	20.5	37.4
		Higher	10.7	9.8	5.9	19.3	54.2
	Cognitive skill	Lower	17.5	12.7	6.9	20.3	42.6
		Higher	13.9	11.2	6.4	19.9	48.6
	Risk tolerance	Aversion	18.2	12.7	6.9	20.1	42.1
		Propensity	12.8	10.6	6.2	19.4	51.1

Looking at each column, we can see which variables most influenced parents’ decision to have their child vaccinated. For example, parents with a secondary education or less have a 36.4% probability of not knowing or refusing to state whether or not they intend to have their child vaccinated. This probability reaches 38.6% if the parent is currently inactive in the labor market. On the other hand, parents with a university education have a likelihood of 53.6% of having their child vaccinated.

The results shown in Table 4 can also be considered line by line. For example, parents who reported a high level of anxiety have a 5.9% probability of belonging to the category “I don’t know or refuse to answer” but a 67.5% probability of answering “Very likely” to have their child vaccinated. Similar differences can be seen when the child has another chronic disease and was vaccinated against influenza in the previous year. Conversely, parents with an education level of “Secondary or less” are 15.1 percentage points more likely to be in the “I don’t know or refuse to answer” category than in the “Very likely” category.

The next recursive model with the ordered probit reverses the direction of causality from parent to child. As shown in Table A1 in the Appendix, some exogenous variables, seen with the SUR specification, explain the parents’ decision to accept a vaccine. Still, the predicted values of their decision in the child equation are not statistically significant. This last result bolsters the reverse causality model running from child to parents presented above.

## Discussion

### Summary of results

The development of a vaccine is an essential step in the effort to end the current COVID-19 pandemic. However, in this study, 19.1% of parents of children with asthma said they were “unlikely” or “very unlikely” to have their child vaccinated if a vaccine was available, and 21.0% said they were “unlikely” or “very unlikely” to get vaccinated themselves. These findings echo growing concern about support for COVID-19 vaccination, even among parents of children with chronic diseases [43, 44].

A major finding of this study emphasizes that at the time of the study, when neither adult nor children COVID-19 vaccination was available, household vaccination decisions revolved more around the child. Our models suggest that once parents decided to have their child vaccinated, there is a high probability that they, too, would agree to get vaccinated. Other studies in health prevention have shown similar results where preventive behaviour decisions centered around the child. For example, for both bicycle helmet use and oral health habits [45], presence of a child in the household helps parents change their own health behaviors.

We found that higher parental educational achievement was associated with a greater intention to have a child vaccinated. This is similar to other reports on vaccination [22, 44] and what has since been documented empirically [21]. Interestingly, this effect is independent and of greater importance than the numeracy level variable or the cognitive test score (“slow” vs. “fast” thinking), which did not emerge as significant predictors. It is possible that this difference in level of education extends beyond education itself and may represent the propensity of some segments of the population to share misinformation [46]. This social contagion effect has also been observed in other vaccine refusal or hesitancy cases clustered either geographically or in specific religious or political groups [47, 48].

Parents who were active in the workforce were more likely to report the intention to vaccinate their child. The considerable potential impact on their income and disruption of their daily lives if they or their child were infected with COVID-19 could be a major factor in their decision [49].

Parents of girls were also more likely to have their child vaccinated. Parents’ perception of the risks of their child being infected varied from 23.2% for a boy to 14.4% for a girl (results not presented). In a study analyzing mothers' responses to sons and daughters engaging in injury-risk behaviors, mothers of daughters intervened more frequently and quickly than mothers of sons and mothers were more tolerant and encouraging of risk taking by sons than by daughters [50]. This may stem from differential risk perception and risk protection behavior from parents between girls and boys [51, 52].

While a child’s asthma control level did not influence the likelihood of parents wanting to have their child vaccinated, parents of a child with another chronic disease were more likely to have their child vaccinated against COVID-19 (predicted probability of “very likely”: 61.6 vs. 43.1%). This result is consistent with the idea that parents of a child with another chronic disease may perceive their child as more fragile and more likely to suffer a severe case of COVID-19, a perception that has since been supported by empirical data on COVID-19 severity in children [27].

We observed a strong correlation between the intention to have a child vaccinated against COVID-19 with a child’s past vaccination with the influenza vaccine. The predicted probability of being “very likely” to have one’s child vaccinated went from 36.0% when the child was not vaccinated against the influenza virus last year to 63.0% when the child was vaccinated. This finding suggests that we can apply (or at least be inspired by) some of the lessons learned from previous studies on the influenza vaccine regarding vaccine acceptance [12, 13].

Finally, similar to studies done with children in general and with children with asthma in particular, contact with a health care provider was a strong predictor of parents’ intent to have their children vaccinated [29]. Despite the diversity of information sources available to families, health professionals are still perceived by many as the most trusted source of health information. This may reflect the fact that those families have had the opportunity to have their concerns heard, their questions answered, or their myths about COVID-19 vaccination dispelled by health care providers. It may also be due to messages being tailored to individual families, leading to greater acceptance of vaccines than can be achieved through general public health messages. These findings underscore the importance of ensuring families have access to health care professionals and of equipping health care workers with the tools required to appropriately inform families and answer their questions and concerns.

### Notable negative findings

This study did not find a significant effect of parental sex, age, or COVID-19-related level of concern. Those variables’ previously documented effects could have been due to other factors of concern, such as parental anxiety level. Perceived risk of infection, numeracy, cognitive reflection test score, and general risk tolerance were also not statistically significant in our study.

Regarding risk tolerance, it is possible that the null impact observed is a combination of opposite effects. Indeed, while risk-averse parents want to have their child vaccinated to avoid the risk of infection, they may also have concerns about a new vaccine and want to avoid the potential risks of vaccination. Parental anxiety may show a similar pattern of mixed-effects leading to a non-statistically significant effect on vaccination intention.

### Limitations

Our study is based on a hypothetical question about a vaccine that was not yet available at the time of the survey in August 2020. Reported intention to vaccinate could (and has likely started to) change as the pandemic continues to evolve, especially as a vaccine for COVID-19 is now available for adults and children > 5 years (no vaccine is yet approved for children under age 5). The once theoretical concern about vaccine acceptance has now become a real public health threat, as COVID-19 cases, hospitalizations, and deaths continue to rise, despite availability of a safe and effective vaccine. More than ever therefore, there is a strong need to educate the general public, actively fight misinformation, and work on public acceptance of a COVID-19 vaccine.

As it has been observed since approval of the vaccine for adults, it is possible that the ongoing and futures wave of the pandemic may shift the risk/benefits balance for some families with regards to vaccination and would affect our estimates, were the study to be conducted now.

Our sample was limited by the size of the number of patients followed in the clinic. It is possible that some factors with small or no effects may have been found to be not statistically significant in this study due to the sample size. Our cross-sectional study can only establish correlation and cannot provide evidence of causation. However, it could still help identify determinants that could influence vaccination decision-making that should be explored.

Our study was also conducted with parents of children with at least one underlying chronic condition (asthma) and many parents with a high education level. However, we think that vaccine acceptance is, if anything, probably higher in this subgroup, given previous reports and our findings that parents of children with other chronic diseases were more likely to report wanting to get vaccinated. As such, 20% of parents unlikely to have their child vaccinated may represent an underestimation at the level of the general population.

## Conclusion

In summary, in this study conducted in August 2020, we have identified important determinants of vaccine intention in parents of children with asthma. Higher parental educational achievement, parents who were active in the workforce, parents of girls, parents of a child with another chronic disease, child’s past vaccination with the influenza vaccine and contact with a health care provider are significantly correlated with parents' intention to have their child with asthma vaccinated against COVID-19.

Now that a vaccine for COVID-19 has become available for adults and children in some jurisdiction (though it has yet to be studied or approved for children under age 5), our result linking parental vaccination first to child welfare and safety might enhance the acceptance of the vaccine by parents.

## Supplementary Information

**Additional file 1:** **Table A1.** Recursive ordered probit models (parents → child).

## Data Availability

Not applicable.

## Footnotes

1 Descriptive statistics suggest that participants choosing the “I don’t know or refuse to answer” category or the “Very unlikely” category share a fair number of sociodemographic characteristics.

2 Confirming the substantive relationship between the two vaccination variables following a Cramer's V coefficient of 0.827.
