# Supplementary material for: Parental decision and intent towards COVID-19 vaccination in children with asthma: an econometric analysis
Source: BMC Public Health. 2022 Aug 13;22:1547. doi: 10.1186/s12889-022-13933-z (PMC9375633; doi:10.1186/s12889-022-13933-z)
Supplement: Supplementary file 1 — Additional file 1: Table A1. Recursive ordered probit models (parents → child). [file 12889_2022_13933_MOESM1_ESM.docx]

**Appendix**

Table A1. Recursive ordered probit models (parents → child)

|  |  |  | Parent’s model | | Child’s model | |
| --- | --- | --- | --- | --- | --- | --- |
|  |  |  | Coef. | p | Coef. | p |
| **Parents' intention to receive vaccine against COVID** (linear prediction) | | | Not included | | -0.864 | 0.771 |
| **Sociodemographic characteristics** | **Sex of parent** (ref.: Male) | Female | 0.361 | 0.218 | 0.731 | 0.49 |
|  | **Age of parent** (ref.: Under 35) | 35 to 44 | 0.119 | 0.433 | 0.147 | 0.71 |
|  |  | 45 and over | -0.122 | 0.535 | -0.278 | 0.499 |
|  | **Level of education** (ref.: Secondary or less) | College (CEGEP) | 0.189 | 0.42 | 0.335 | 0.587 |
|  |  | University | 0.576 | 0.001 | 1.056 | 0.532 |
|  | **Employment status** (ref.: Inactive) | Active | 0.507 | 0.003 | 1.038 | 0.484 |
|  | **Region of residence** (ref.: Other region) | Montreal | 0.208 | 0.128 | 0.399 | 0.535 |
|  | **Sex of child** (ref.: Male) | Female | Not included | | 0.341 | 0.019 |
|  | **Age of child** (ref.: 5 and under) | 6 to 10 | Not included | | 0.03 | 0.867 |
|  |  | 11 and over |  |  | 0.37 | 0.097 |
| **Clinical characteristics** | **Perceived control of child’s asthma** (ref.: Less controlled) | More controlled | Not included | | 0.067 | 0.744 |
|  | **Child has another chronic disease** (ref.: No) | Yes | Not included | | 0.571 | 0.028 |
|  | **Child was vaccinated against influenza last year** (ref.: No) | Yes | Not included | | 0.795 | < 0.001 |
|  | **Consultation with a health professional** (ref.: No) | Yes | Not included | | 0.404 | 0.009 |
| **Risk perception** | **Level of general anxiety** (ref.: Lower) | Average | -0.025 | 0.883 | -0.059 | 0.758 |
|  |  | Higher | 0.382 | 0.115 | 0.818 | 0.48 |
|  | **Level of concern regarding COVID-19** (ref.: Lower) | Average | 0.335 | 0.099 | 0.604 | 0.547 |
|  |  | Higher | 0.354 | 0.082 | 0.394 | 0.704 |
|  | **Know someone that has been affected by COVID-19** (ref.: No) | Yes | -0.048 | 0.723 | Not included | |
|  | **Perceived control of child’s risk of infection with COVID-19** (ref.: Less controlled) | More controlled | Not included | | 0.029 | 0.837 |
|  | **Perceived risks of infection with COVID-19** (ref.: 50%) | Less than 50% | Not included | | 0.209 | 0.189 |
| **Cognitive characteristics** | **Numeracy level** (ref.: Lower) | Higher | 0.356 | 0.023 | 0.516 | 0.627 |
|  | **Cognitive skill** (ref.: Lower) | Higher | 0.128 | 0.466 | 0.185 | 0.644 |
|  | **Risk tolerance** (ref.: Aversion) | Propensity | 0.082 | 0.59 | 0.28 | 0.333 |
| Log pseudolikelihood | | | -404 | | -384 | |
| Pseudo R2 | | | 0.062 | | 0.114 | |
